# ATBF1 is a potential diagnostic marker of histological grade and functions via WNT5A in breast cancer

**DOI:** 10.1186/s12885-022-10380-2

**Published:** 2022-12-07

**Authors:** Mei Li, Yanan Zheng, Xujun Li, Xiaohan Shen, Tingxia Zhang, Bowen Weng, Haijiao Mao, Jiyuan Zhao

**Affiliations:** 1 Zhejiang Key Laboratory of Pathophysiology, School of Medicine, Ningbo University, 818 Fenghua Road, Ningbo, Zhejiang 315211 China; 2 Ningbo Institute of Medical Sciences, Ningbo, Zhejiang China; 3 Department of Breast Surgery, Hwa Mei Hospital, University of Chinese Academy of Sciences, Ningbo, Zhejiang China; 4 Ningbo Diagnostic Pathology Center, Ningbo, Zhejiang China; 5 Department of Orthopaedic Surgery, the Affiliated Hospital of Medical School, Ningbo University, Ningbo, Zhejiang China

**Keywords:** ATBF1, Mislocalization, Histological grade, Cell differentiation, WNT5A, Breast cancer

## Abstract

**Background:**

Histological grade has been demonstrated to be an important factor of breast cancer outcome and is associated with cell differentiation and is currently being evaluated via H&E-stained sections. Molecular biomarkers are essential to improve the accuracy of histological grading. ATBF1, a large transcription factor, has been considered a tumor suppressor gene with frequent mutations or deletions in multiple cancers. In breast cancer, ATBF1 was reported to function in cell differentiation and mammary development. However, its role in the clinic has rarely been reported.

**Methods:**

Breast cancer tissues (BCTs) and adjacent noncancerous tissues (ANCTs) were collected to analyze the expression of ATBF1 at the mRNA and protein levels. Three anti-ATBF1 antibodies recognizing independent peptides of ATBF1 (N-terminal end, middle region and C-terminal end) were applied for IHC staining. Small interfering RNA (siRNA) was used to silence ATBF1 expression and to investigate the roles of ATBF1 in MCF7 cells. Microarrays were introduced to analyze the differentially expressed genes, enriched GO terms and KEGG terms regulated by ATBF1 and its potential downstream genes, which were further confirmed in vitro and in clinical samples.

**Results:**

The expression of ATBF1 was reduced in BCTs at both the mRNA and protein levels compared with that in ANCTs. ATBF1 protein was predominantly localized in the nucleus of ANCTs but in the cytoplasm of BCTs. Both the mRNA and protein levels of ATBF1 were significantly correlated with histological grade. Consistently, knockdown of ATBF1 increased stemness marker expression and reduced differentiation markers in vitro. Further analysis identified WNT5A as an essential downstream gene of ATBF1 in breast cancer cells. Treatment of WNT5A disrupted cell proliferation induced by ATBF1 silencing. In BCTs, a significant correlation was observed between the expression of WNT5A and ATBF1.

**Conclusion:**

The results indicated that ATBF1 expression might be a useful diagnostic marker associated with histological grade and breast cancer malignancy. WNT5A and its signaling pathway are novel mechanisms by which ATBF1 contributes to breast cancer tumorigenesis.

**Supplementary Information:**

The online version contains supplementary material available at 10.1186/s12885-022-10380-2.

## Introduction

AT motif-binding factor 1 (ATBF1), also known as zinc finger homeobox 3 (ZFHX3), is a large transcription factor composed of 3,703 amino acid residues with a molecular weight of 404kD. It contains four homeodomains and 23 zinc finger motifs that regulate the transcription of target genes by binding to their promoters [1]. ATBF1 is reported to play roles in multiple pathophysiological processes, especially cell differentiation (such as neuronal differentiation [2], myogenic differentiation [3] and embryonic differentiation [4]) and carcinogenesis. Increasing evidence has demonstrated ATBF1 as a strong candidate tumor suppressor gene in multiple tumors, including prostate cancer [1, 5], gastric cancer [6], liver cancer [7], colon cancer [8] and non-small cell lung cancer [9]. In prostate cancer, the chromosomal locus of ATBF1 was frequently found to be deleted, and somatic mutations were also frequently identified, which impaired ATBF1 function [10]. Downregulation of ATBF1 was correlated with worse patient survival [10]. Recent studies further found that ATBF1 inhibited prostate cancer cell proliferation via cooperation with ESR2 to regulate the transcription of MYC [1]. Higher levels of ATBF1 and estrogen receptor 2 (ESR2) in prostate cancer tissue samples were correlated with better patient survival [1]. In addition toATBF1 expression levels, mislocalization of ATBF1 from the nucleus to the cytoplasm was proven to be correlated with cell differentiation and histopathologic progression (including cancer metastasis) in head and neck squamous cell carcinoma [11], colon cancer [8], skin cancer [12] and bladder carcinoma [13].

Recently, accumulated evidence has demonstrated the role of ATBF1 in breast epithelial cell differentiation, mammary gland development and breast cancer tumorigenesis via its participation in multiple hormone-hormone receptor signaling pathways [14–17]. In estrogen receptor (ER)-positive cells, ATBF1 inhibited ER function by selectively competing with AIB1 for binding to the ER [18]. Meanwhile, estrogen upregulated ATBF1 transcription but caused its protein degradation through the E3 ubiquitin ligase EFP [19, 20]. ATBF1 likely regulates pubertal mammary gland development by inhibiting the proproliferative function of the estrogen-ER signaling pathway [14], while its expression is essential for progesterone-progesterone receptor (PR) signaling [17] and prolactin-prolactin receptor (PrlR) signaling [16] to drive ductal side branching and alveologenesis. Moreover, SUMOylation of ATBF1 at Lys-2806 enhanced its stability and function in cell proliferation in breast cancer cells [21]. In breast cancer patients, although infrequent mutations occur, the chromosomal locus of ATBF1 is deleted in as many as 75% of ductal cancers and 100% of lobular cancers [22, 23]. *ATBF1-A* mRNA expression was reported to be significantly associated with tumor size, lymph node metastasis, estrogen receptor (ER) and breast cancer prognosis [24]. However, the protein level and localization of ATBF1 in breast cancer and the relationship between ATBF1 protein and clinical indices of breast cancer have rarely been reported. It is also not clear whether ATBF1 plays roles in breast cancer by regulating signaling pathways other than hormonal signaling pathways.

The aim of the present study was to clarify the correlation between ATBF1 expression and breast cancer in clinical samples, to discover the functions of ATBF1 in breast cancer cells and to investigate the potential mechanism of ATBF1 in breast cancer. Three anti-ATBF1 antibodies recognizing different regions were applied to assess the significance of ATBF1 protein in breast cancer diagnosis. Transcriptomics was used to explore the overall differentially expressed genes and the potential GO and KEGG pathways affected by ATBF1. Following bioinformatics analysis, WNT5A was predicted to be an essential downstream gene of ATBF1. The expression and function of WNT5A regulated by ATBF1 was further investigated in breast cancer cell lines and clinical samples.

## Materials and methods

### Reagents, cells and patient samples

**Fig. 1:**
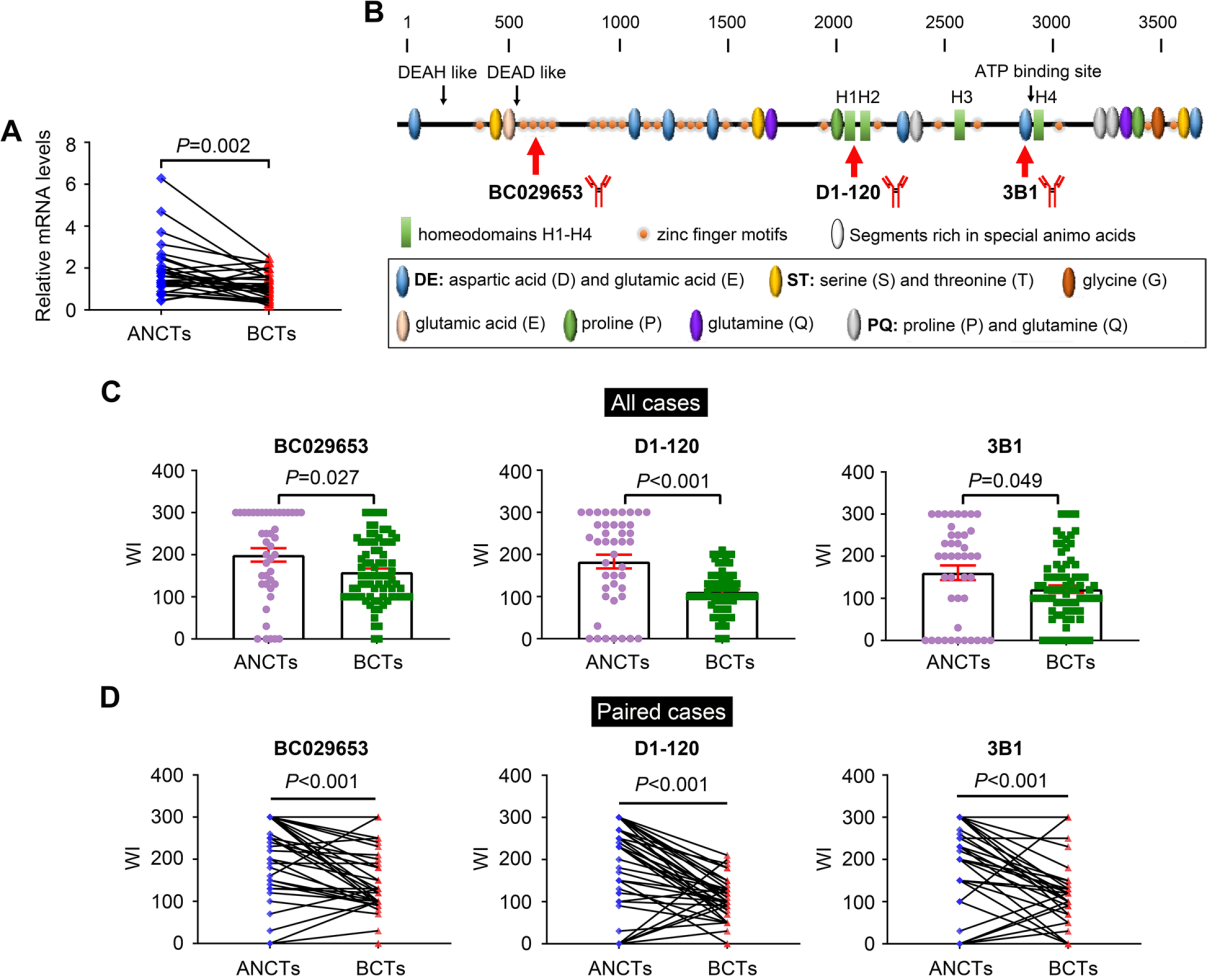
The expression of ATBF1 was reduced in breast cancer tissues (BCTs) compared with that in adjacent noncancerous tissues (ANCTs) at both mRNA and protein levels. **A** The mRNA levels of ATBF1 in paired ANCTs and BCTs (*n* = 31) as determined by real-time qPCR. **B** Molecular structure of ATBF1 and the respective recognition site of three anti-ATBF1 antibodies.BC029653 recognizes the amino acids from 517 to 787; D1-120 recognizes the amino acids from 2107 to 2147; 3B1 recognizes the amino acids from 2811 to 2910. **C** The protein levels of ATBF1 in all cases (43 ANCTs and 80 BCTs as a pool respectively). **D** The protein levels of ATBF1 in paired ANCTs and BCTs (*n* = 43). The expression of ATBF1 protein were determined by IHC staining. Weight index (WI) was used to quantify the protein levels of ATBF1 by multiplying staining intensity by the percentage of positive cells, as previously reported [11]. *P*-value was calculated by student’s *t* test under paired (A &D) or unpaired (C)

The reagents were purchased from their respective vendors: expose mouse- and rabbit-specific HRP/DAB detection IHC kit (Cat. ab236466, Abcam, Shanghai, China); RNA-Solv Reagent (Cat. R6830, Omega, Guangzhou, China); TRIzol™ (Cat. 15,596,026, Invitrogen, Shanghai, China); BCA Protein Assay Kit (Cat. CW0014S, CWBIO, Jiangsu, China); secondary antibodies (BOSTER, Wuhan, China); Lipofectamine RNAiMAX reagent (Cat. 13,778,100, Invitrogen); recombinant human WNT5A (Cat. CSB-EP026138HU1, CUSABIO, Wuhan, China); cell counting kit (APE×Bio, Shanghai, China); WNT5A primary antibody (Cat. A19133, Abclonal, Wuhan, China); and β-actin primary antibody (Cat. AC026, Abclonal). ATBF1 primary antibodies were designed to recognize independent peptides, as shown in Fig. 1B: BC029653 (Abcam, Shanghai, China), which recognizes the N-terminus of ATBF1 (amino acids, 517–787); D1-120 (MBL, Shanghai, China), which recognizes a middle region of ATBF1 (amino acids, 2107–2147); and 3B1 (Santa Cruz Biotechnology, Shanghai, China), which recognizes the C-terminus of ATBF1 (amino acids, 2811–2910). Primers for mRNA expression analysis were synthesized by Shanghai Generay Biotech Co., Ltd., and the primer sequences are listed in Table 1. Small interfering RNAs (siRNAs) were synthesized by Shanghai GenePharma Co., Ltd. The sequence of siATBF1 is 5′-AGAAUAUCCUGCUAGUACA-3′, as previously reported [25].

**Table 1:** Primer sequences for real-time qPCR amplification

Genes		Primer sequences (5’-3’)	Length (bps)
ATBF1	Forward	TGTTCCAGATCGAGATGGGAAT	76
	Reverse	CTTTCCCAGATCCTCTGAGGTTT	
WNT5A	Forward	AAGTTGGTACAGGTCAACAGCCGCT	170
	Reverse	CACATGAGCTCGCAGCCATCCATG	
EpCAM	Forward	GCCAAATGTTTGGTGATGAA	110
	Reverse	TCGCAGTCAGGATCATAAAG	
CK7	Forward	TGAAATTAACCGCCGCACAG	278
	Reverse	TGCATTTGGCCATCTCCTCA	
CK18	Forward	CCTACAAGCCCAGATTGCCA	115
	Reverse	CCGAGCCAGCTCGTCATATT	
GAPDH	Forward	GGTGGTCTCCTCTGACTTCAACA	127
	Reverse	GTTGCTGTAGCCAAATTCGTTGT	

Breast cancer cells (MCF7) were purchased from the Type Culture of the Chinese Academy of Sciences (Shanghai, China) and cultured in MEM supplemented with 10% fetal bovine serum (FBS) in a 37 °C in a 5% CO_2_ humidified incubator.

A total of 44 fresh-frozen samples from breast cancer tissues (BCTs) and 31 paired adjacent noncancerous tissues (ANCTs) were collected from the Department of Breast Surgery in Hwa Mei Hospital (Ningbo, China) between 2018 and 2019. The fresh samples were stored in liquid nitrogen for mRNA isolation and qPCR analysis. Then, a total of 80 formalin-fixed and paraffin-embedded BCTs and 43 paired ANCTs collected from Ningbo Diagnostic Pathology Center between 2011 and 2013 were collected for immunohistochemistry (IHC) staining. After the initial surgery, none of the patients received chemotherapy or radiation therapy.

### IHC staining

The paraffin-embedded tissue sections were deparaffinized and rehydrated following the standard procedure. Sections were incubated with 3% H_2_O_2_ to block endogenous peroxidase activity, and antigen retrieval was performed in citrated buffer at 110 °C in a pressure cooker. Then, the sections were incubated with the three anti-ATBF1 antibodies at 4 °C overnight. Antigen retrieval time and antibody dilutions were optimized as follows: BC029653 for ATBF1 (7 min, 1:200), D1-120 for ATBF1 (5 min, 1:3000), and 3B1 for ATBF1 (5 min, 1:50) and WNT5A (5 min, 1:100). The following procedures were performed using an expose mouse- and rabbit-specific HRP/DAB detection IHC kit according to the manufacturer’s instructions. After application of DAB-chromogen, the immune complexes were visualized as brown to show the protein levels and localization. Nuclei were counterstained with hematoxylin, and the slides were dehydrated, mounted and analyzed with a light microscope. Staining of all tissue sections was performed under nearly identical conditions.

Both ANCTs and BCTs were scored for ATBF1 and WNT5A protein expression. Moreover, the protein levels of ATBF1 in the nucleus and cytoplasm were assessed separately. The intensity of IHC staining was measured using a numerical scale (0 = no staining, 1 = weak staining, 2 = moderate staining, and 3 = strong staining) as previously reported [11]. The percentage of positive cells was also assessed. The weight index (WI) was calculated by multiplying the staining intensity by the percentage of positive cells.

### RNA isolation and real-time qPCR amplification

In this study, mRNA was isolated from cultured cells and breast tissues. The cultured cells were rinsed with PBS and lysed in RNA-Solv Reagent according to the manufacturer’s instructions. The tissue samples were sheared with scissors and ground with an electric grinder. The mRNA from breast tissues was extracted using TRIzol™ following the manufacturer’s instructions. The concentration and purity of mRNA were assessed by UV absorbance at 230 nm, 260 nm, and 280 nm using a microplate spectrophotometer. First-strand cDNA was synthesized using TransScript® One-Step gDNA Removal and cDNA Synthesis SuperMix. Real-time qPCR was performed as previously reported [26]. GAPDH was used as an internal control.

### Protein extraction and western blotting

Cells were rinsed with PBS and lysed in cold RIPA buffer containing protease and phosphatase inhibitors (PMSFs) at 4 °C for 30 min. Lysates were harvested physically with a cell scraper and centrifuged for 10 min at 4 °C and 10,000 rpm. The supernatant was collected, and the protein concentration was determined using a BCA Protein Assay Kit. Protein samples were prepared by adding loading buffer, heating the samples at 98 °C for 5 min, and then cooling them on ice for 2–3 min. WNT5A and β-actin were separated by 10% sodium dodecyl sulfate‒polyacrylamide gel electrophoresis (SDS‒PAGE) and transferred to nitrocellulose membranes. The membranes were blocked in 5% nonfat powdered skim milk for 1 h, followed by primary antibody (WNT5A, 1:1000; β-actin, 1:10000) incubation at 4 °C overnight. After washing with TBS-T, the secondary antibodies were applied for 1 h at room temperature. The chemiluminescent signal was visualized with an optical microscope (Clinx, Shanghai, China). Original blots of WNT5A were provided in supplementary materials.

### RNA interference (RNAi)

Synthesized siRNA was used to knockdown the expression of ATBF1. MCF7 cells were trypsinized into single cells and seeded on a 24-well plate at a density of 3 × 10^4^ cells in complete medium. Sixteen hours later, the cells with a confluence of 30–50% were starved in 500 µL MEM medium for 2 h. Lipofectamine RNAiMAX was introduced to enhance the transfection efficiency of siATBF1 into MCF7 cells according to the manufacturer’s instructions, as previously reported [25]. Briefly, siRNAs and RNAiMAX reagent were separately diluted in 50 µL MEM medium with gentle mixing. Five minutes later, the diluted siRNA and lipid reagent were combined together, gently mixed, and incubated for another 15–20 min at room temperature. The mixture was added to each well containing cells. The cells were cultured with fresh complete medium after 6–8 h for the desired time.

### Cell proliferation assay

After 24 h of siRNA transfection, cells were treated with 200 ng/mL recombinant human WNT5A to rescue the downregulation of WNT5A induced by ATBF1 knockdown and to assess the further effect on cell proliferation. The cell number was determined after 24 h of WNT5A treatment with a cell counting kit according to the manufacturer’s instructions. Briefly, fresh medium (360 µl) with CCK reagent (40 µl) was added to each well containing cells, followed by incubation at 37 °C for 2 h. The solution (100 µl) from each well was transferred to a 96-well plate, and absorbance at 450 nm was measured using a microplate reader.

### mRNA microarray analysis

RNA sequencing and bioinformatics analysis of mRNA microarray data were performed by Shanghai Applied Protein Technology. Briefly, total RNA was extracted from MCF7 cells transfected with siControl or siATBF1 (n = 3 per group) using TRIzol™ Reagent. RNA samples were quantified based on the A260/A280 absorbance ratio and the RIN. Only quantified samples were used for library construction. Paired-end libraries were prepared using an ABclonal mRNA-seq Lib Prep Kit (ABclonal, China) according to the manufacturer’s instructions. The mRNA was purified from total RNA with oligo (dT) magnetic beads, followed by the synthesis of first-strand cDNA with random primers using mRNA fragments as templates. The second-strand cDNA was synthesized using DNA polymerase. The paired-end library was prepared with adaptor-ligated double-stranded cDNA. Finally, sequencing was performed with an Illumina Novaseq 6000/MGISEQ-T7 instrument.

The data generated from the Illumina/BGI platform were used for bioinformatics analysis. Raw data in fastq format were first processed through in-house Perl scripts. The adaptor sequence was removed, and low quality reads with a string quality value less than or equal to 25, accounting for more than 60% of all reads, were also filtered out. Then, the clean reads were separately aligned to the human genome with orientation mode using HISAT2 software to obtain mapped reads. Feature counts were used to count the read numbers mapped to each gene. The FPKM of each gene was then calculated based on the length of the gene and the read count mapped to the gene. Differential gene expression was analyzed using DESeq2, and genes with |log2FC|>1 and *P*adj < 0.05 were considered to be significantly differentially expressed genes. The GO and KEGG enrichment analyses of differentially expressed genes were performed using the cluster Profiler R software package, which can be used to explain the functional enrichment of differentially expressed genes and to clarify the differences between samples at the gene function level. The GO and KEGG terms were considered significantly enriched when *P* < 0.05.

### Statistical analysis

Statistical analyses were performed using SPSS® statistical software (SPSS Inc., Chicago, IL, USA). Student’s *t* test was used to determine significant differences between two groups. A paired *t* test was used to determine significant differences between paired tissues (ANCTs and BCTs). One-way ANOVA with Bonferroni correction was used to determine significant differences among the three groups. *P* values less than 0.05 were considered statistically significant.

## Results

### Expression of ATBF1 in breast cancer tissues

ATBF1 expression in breast cancer tissues (BCTs) was assessed at both the mRNA and protein levels. As shown in Fig. 1A, the mRNA levels of *ATBF1* in BCTs were significantly lower than those in paired adjacent noncancerous tissues (ANCTs). IHC staining was performed in 80 BCTs and 43 ANCTs to examine ATBF1 protein expression and localization. A schematic diagram of the ATBF1 structure and three anti-ATBF1 antibodies recognizing independent peptides (BC029653 (517–787 AA), D1-120 (2107–2147 AA), and 3B1 (2811–2910 AA)) are shown in Fig. 1B. Consistent with the mRNA levels of *ATBF1* in BCTs, the expression of ATBF1 protein was reduced in BCTs compared with that in ANCTs in all cases (Fig. 1C), which was significant with all three anti-ATBF1 antibodies. The downregulation of ATBF1 was more apparent in paired cases with lower *P* values than in all cases. Thus, ATBF1 expression was significantly reduced in cancer tissues at both the mRNA and protein levels, which has also been observed in other cancer types, such as prostate cancer [10], colorectal cancer [27] and hepatocellular carcinoma [28].

**Fig. 2:**
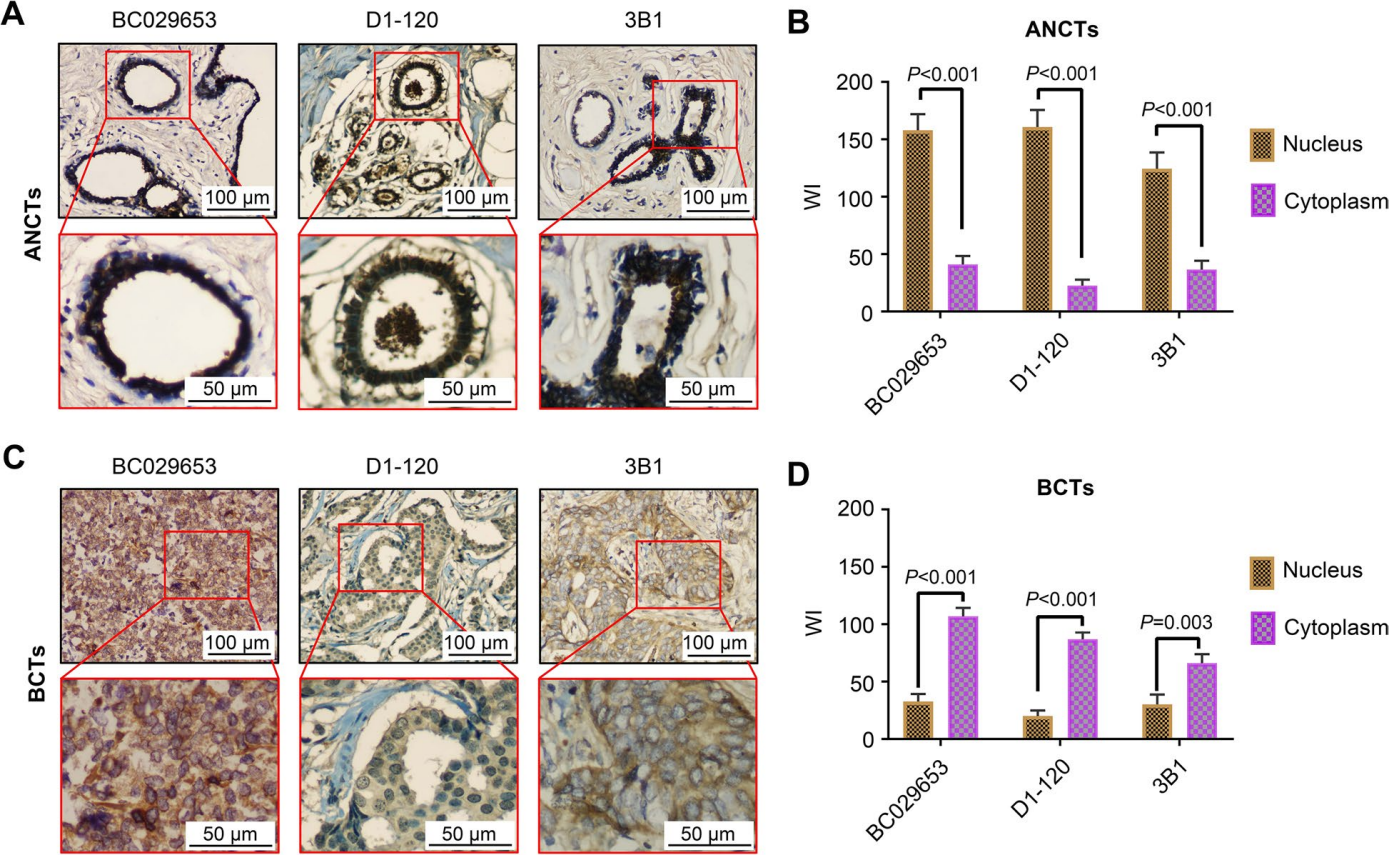
Subcellular localization of ATBF1 in breast cancer patients as determined by IHC staining. **A**-**B** ATBF1 protein was mainly localized in the nucleus in ANCTs. **C**-**D** ATBF1 protein was mainly localized in the cytoplasm in BCTs. (A&C) Representative IHC images of the three ATBF1 antibodies. (B&D) Quantification of ATBF1 staining in the nucleus and cytoplasm of the paired cases (*n* = 43), as determined by WI index. *P*-value between two groups was calculated by student’s *t* test. Error bars represent the standard deviation of the mean

In addition to lower expression levels, mislocalization of ATBF1 was also observed in BCTs based on IHC staining (Fig. 2). The staining signals in both the cytoplasm and nucleus in each case were scored separately. ATBF1, as a transcription factor, was mainly located in the nucleus in most normal tissues (Fig. 2A&B), while significant cytoplasmic staining was observed in BCTs (Fig. 2C&D). The mean WI of nuclear ATBF1 in BCTs (BC029653, WI = 32.79 ± 6.40; D1-120, WI = 20.00 ± 4.90; 3B1, WI = 30.23 ± 8.53) was lower than that in ANCTs (BC029653, WI = 168.60 ± 13.90; D1-120, WI = 166.74 ± 15.75; 3B1, WI = 126.74 ± 15.37). Meanwhile, the mean WI of cytoplasmic ATBF1 in BCTs (BC029653, WI = 106.98 ± 7.29; D1-120, WI = 86.98 ± 5.89; 3B1, WI = 66.05 ± 7.81) was higher than that in ANCTs (BC029653, WI = 50.23 ± 8.51; D1-120, WI = 31.16 ± 6.27; 3B1, WI = 44.88 ± 9.11).

### Correlation of ATBF1 expression with clinical characteristics

The patient and tumor characteristics for *ATBF1* mRNA analysis are summarized in Table 2 (*n* = 44). The age of the patients ranged from 36 to 80 years, and the median age at diagnosis was 63 years. Patients were separated into two groups by tumor size: > 2 cm (25 cases) and ≤ 2 cm (19 cases), and the median tumor size was 2.57 cm. Regional lymph node involvement was identified in 20 cases. Stage I and II patients were grouped together (25 cases), and the remaining 19 cases were stage III. The clinical pathological parameters of molecular receptors (including ER, PR and HER2) were also considered. More cases were diagnosed as ER+ (28 cases, 63.6%), PR+ (29 cases, 65.9%) or HER2+ (30 cases, 68.2%) than ER-, PR-, or HER2-. The expression of ER and PR exhibited the same trend (-/+) in more than 93% of cases. The results showed that *ATBF1* mRNA levels were only significantly associated with histological grade (*P* = 0.04) but were not associated with tumor size, lymph node metastasis (LNM), or ER, PR or HER2 status. A negative correlation was observed between *ATBF1* mRNA levels and histological grade, while higher *ATBF1* mRNA levels were detected in lower grade tumors.

**Table 2:** The correlation between ATBF1 mRNA levels andclinicopathological parameters

Characteristics	Relative mRNA expression ofATBF1(SE)	*P* value
Tumor size		
≤2(19)	1.51(0.19)	0.31
>2(25)	1.26(0.15)	
LNM		
Yes(20)	1.30(0.18)	0.60
No(24)	1.43(0.16)	
Grade		
I-II(25)	1.58(0.16)	0.04*
III(19)	1.09(0.15)	
ER		
(-)(16)	1.12(0.19)	0.13
(+)(28)	1.51(0.16)	
PR		
(-)(15)	1.12(0.16)	0.13
(+)(29)	1.50(0.17)	
HER2		
(-)(14)	1.51(0.29)	0.51
(+)(30)	1.30(0.11)	

*Abbreviations*: *SE* standard error, *LNM* lymph node metastasis, *ER* estrogen receptor, *PR* progesterone receptor, *HER2* human epidermal growth factor 2

*P < 0.05

**Fig. 3:**
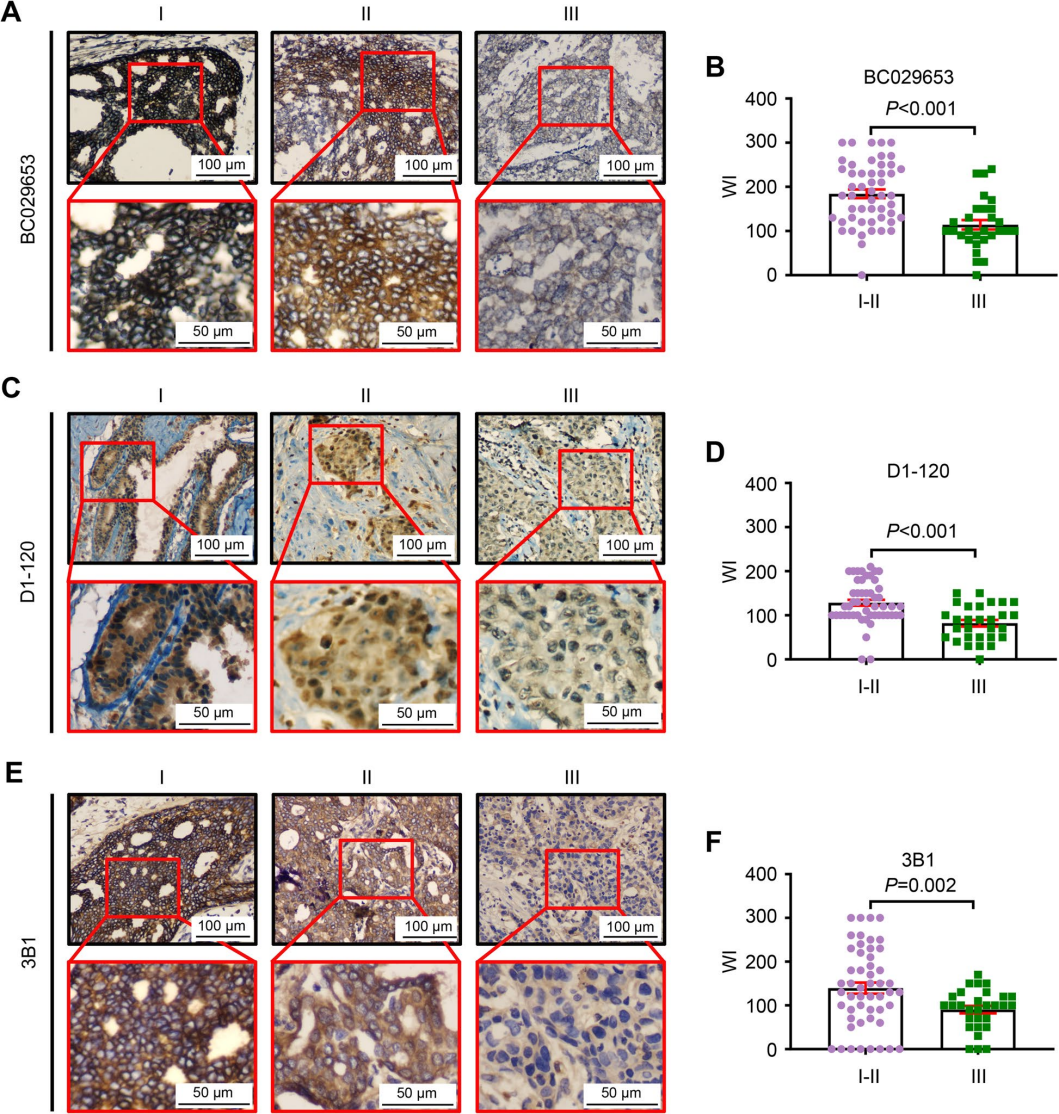
The expression of ATBF1 protein was correlated with histological grade. The ATBF1 protein levels were determined by IHC staining with the three independent antibodies, BC029653 (**A**&**B**), D1-120 (**C**&**D**) and 3B1 (**E**&**F**). Representative IHC images of ATBF1 staining at different histological grade were shown (A, C&E). ATBF1 protein levels were quantified by WI index (B, D&F). The expression of ATBF1 was significantly down-regulated in higher stages than that in lower stages as detected by all three antibodies. *P*-value between two groups was calculated by student’s *t* test

Meanwhile, the correlation between ATBF1 protein and clinical pathological parameters was also analyzed in 80 BCTs with all three anti-ATBF1 antibodies. The tumor characteristics used for ATBF1 protein analysis are summarized in Table 3, and are the same as those in Table 2. Interestingly, the ATBF1 protein expression levels measured by the anti-N-terminal antibody BC029653 and the anti-C-terminal antibody 3B1, but not the anti-middle antibody D1-120, were significantly correlated with tumor size. Consistent with the mRNA results, ATBF1 protein levels were also significantly negatively correlated with histological grade (BC029653, *P* < 0.001; D1-120, *P* < 0.001; 3B1, *P* = 0.002). Representative IHC staining images and columnar statistical charts of BCTs stained with three anti-ATBF1 antibodies at different histological grades are shown in Fig. 3.

**Table 3:** The correlation between ATBF1 protein levels and clinicopathological parameters

Characteristics	ATBF1 WI(SE)					
	BC029653	*P* value	D1-120	*P* value	3B1	*P* value
Tumor size						
≤2(41)	177.3(11.3)	0.02*	117.1(8.8)	0.32	151.0(13.7)	0.001*
>2(39)	139.5(11.6)		105.9(6.8)		91.0(9.6)	
LNM						
Yes(36)	157.2(10.6)	0.86	110.6.4(7.4)	0.73	109.4(10.8)	0.22
No(44)	160.2(12.5)		113.4(8.3)		131.8(13.7)	
Grade						
I-II(51)	184.3(9.9)	<0.001*	127.1(6.4)	<0.001*	138.6(12.7)	0.002*
III(29)	114.3(10.7)		82.1(7.4)		90.3(8.5)	
ER						
(-)(27)	143.3(13.9)	0.16	105.6(9.8)	0.44	102.2(15.5)	0.16
(+)(53)	168.1(10.2)		114.7(6.9)		128.5(10.4)	
PR						
(-)(30)	135.0(13.5)	0.03*	99.7(8.4)	0.10	104.7(13.4)	0.17
(+)(50)	173.2(10.1)		118.8(7.3)		129.8(11.7)	
HER2						
(-)(35)	150.6(14.4)	0.40	110.0(8.8)	0.80	116.6(13.7)	0.45
(+)(45)	165.3(9.6)		112.9(7.3)		130.2(11.8)	

*P < 0.05

Histological grade is usually associated with the differentiation status of cancer cells, and a higher grade corresponds with less differentiated carcinomas, which indicates that ATBF1 expression might be associated with cell differentiation and may influence tumor malignancy.

### The relationship between ATBF1 and cell differentiation in vitro

**Fig. 4:**
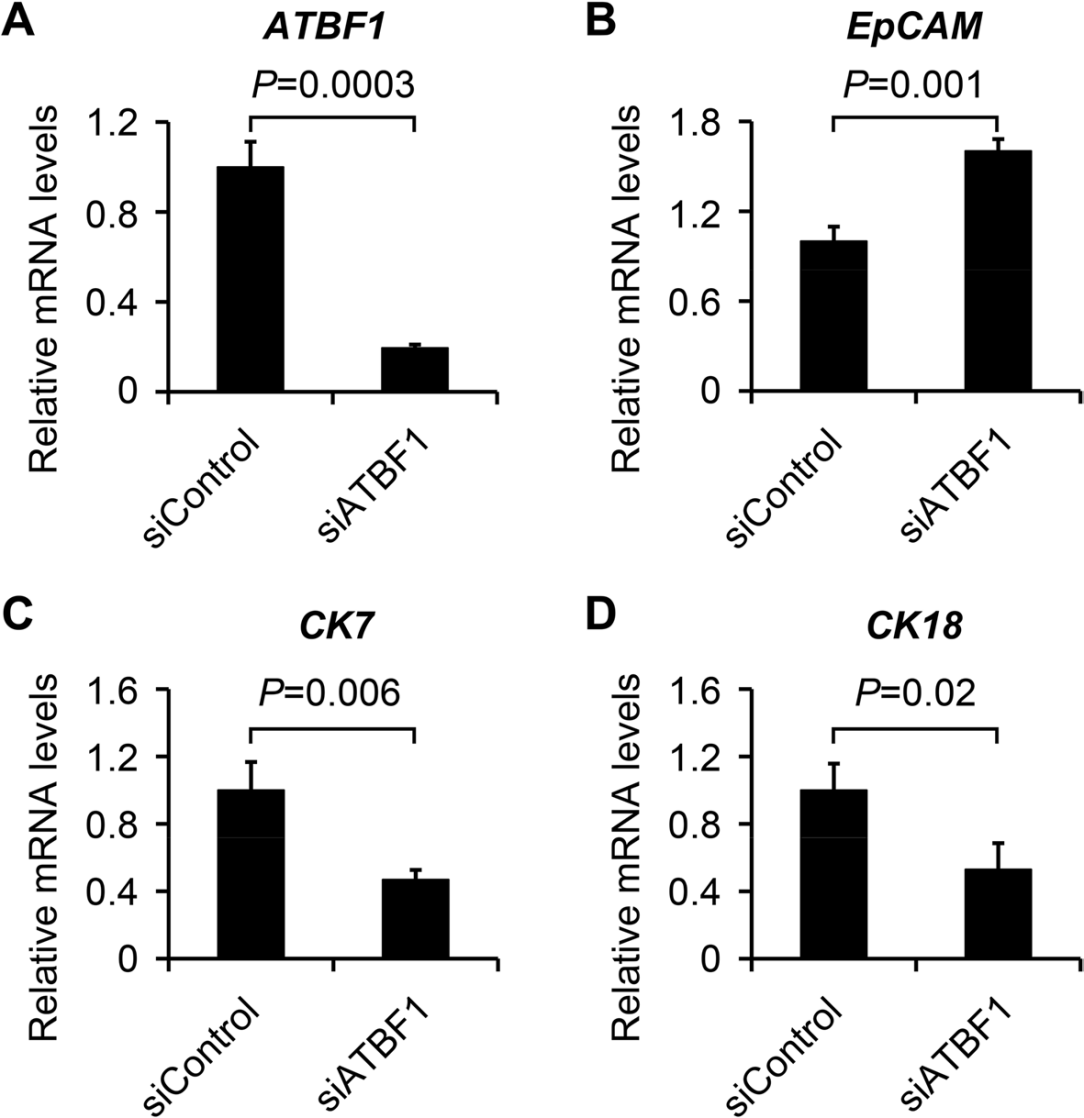
Knockdown of ATBF1 by siRNA inhibited differentiation of breast cancer cells. MCF7 cells were seeded on 24-well plates, and the confluence was around 30% after 16 h of seeding. ATBF1 siRNA (siATBF1) or control siRNA (siControl) were transfected into MCF7 cells with Lipofectamine RNAiMAX at the final concentration of 150 nM. The mRNA was extracted for PCR amplification after 24 h of transfection. The silencing efficiency of *ATBF1* (**A**) and differentiation associated genes (*EpCAM* (**B**), *CK7* (**C**) and *CK18* (**D**)) was determined by real-time qPCR. *EpCAM* is a stemness marker, while *CK7* and *CK18* are cell differentiation markers. Triplicate experiments were performed and *P*-value between two groups was calculated by student’s *t* test

To further confirm the relationship between ATBF1 and cell differentiation, we knocked down the expression of ATBF1 with siRNA in MCF7 cells, which express high levels of ATBF1 compared to normal breast cancer cells. After siATBF1 transfection, more than 70% of *ATBF1* mRNA was silenced (Fig. 4A), which was consistent with our previous studies [14, 25]. Three genes were examined to assess the effect of ATBF1 on cell differentiation, one stemness-associated gene (EpCAM) and two differentiated genes (CK7 and CK18). *EpCAM* mRNA levels were higher in the siATBF1 group than in the siControl group (Fig. 4B), while CK7 and CK18 mRNA levels were lower in the siATBF1 group than in the siControl group (Fig. 4C-D). The results indicated that downregulation of ATBF1 may inhibit breast cancer cell differentiation. The results were also consistent with a clinical study showing lower ATBF1 expression was associated with higher histological grade, lower differentiated status and higher malignancy of breast cancer.

### Microarray analysis of ATBF1-related mRNA expression profiles

**Fig. 5:**
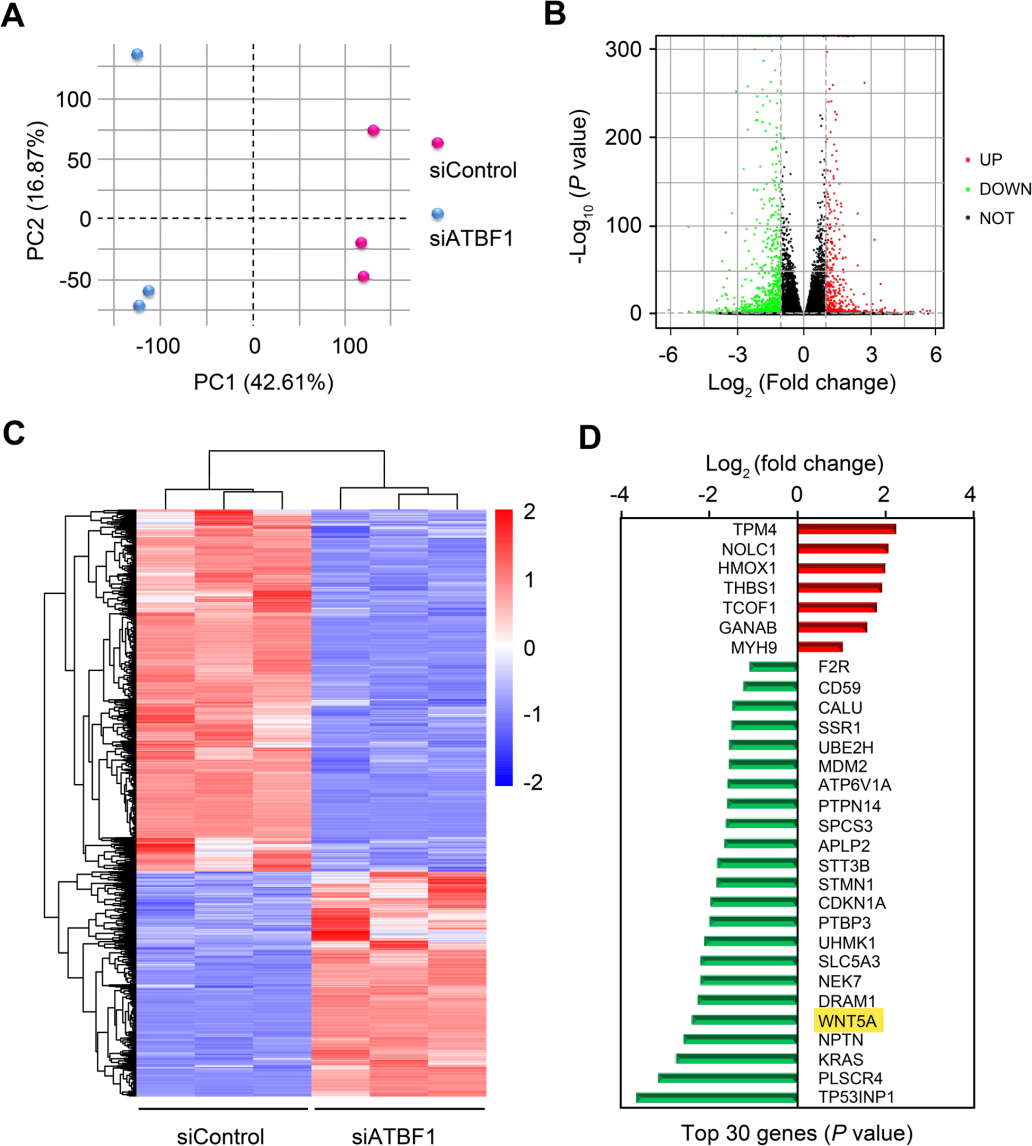
Differentially expressed genes regulated by ATBF1were analyzed by microarray and bioinformatics assay. MCF7 cells were transfected with siControl or siATBF1 at the concentration of 150 nM. The total mRNA were extracted for microarray analysis after 48 h. **A** Two-dimensional principal component analysis (PCA) plot. **B** Volcano map. Green dots, significantly down-regulated genes; red dots, significantly up-regulated genes; black dots, genes with no significant difference. **C** Hierarchical cluster of differentially expressed genes. The distances of the gene branches in the left dendrogram represent the similarity of the expression levels. **D** Top 30 genes regulated by ATBF1 were ranked with *P* value. Red, up-regulated genes; green, down-regulated genes

To further investigate the potential molecular mechanism of ATBF1 in breast cancer tumorigenesis, mRNA microarray analysis was performed to identify ATBF1-related mRNA expression profiles in MCF7 cells. The expression distributions in the siControl group and siATBF1 group were different, as observed via principal component analysis (PCA) (Fig. 5A). According to microarray expression profiling data, 58 967 mRNAs were detected, and 1713 mRNAs were differentially expressed between the two groups, with 658 upregulated genes and 1055 downregulated genes in the siATBF1 group compared to thesiControl group (Fig. 5B&C). mRNA expression variation was assessed by scatter plot (Fig. 5B), while hierarchical clustering was used to show the mRNA expression patterns (Fig. 5C). The top 30 DEGs with the highest *P* value are listed in Fig. 5D. The pattern was similar to that of all differentially expressed genes, with more downregulated genes than upregulated genes.

**Fig. 6:**
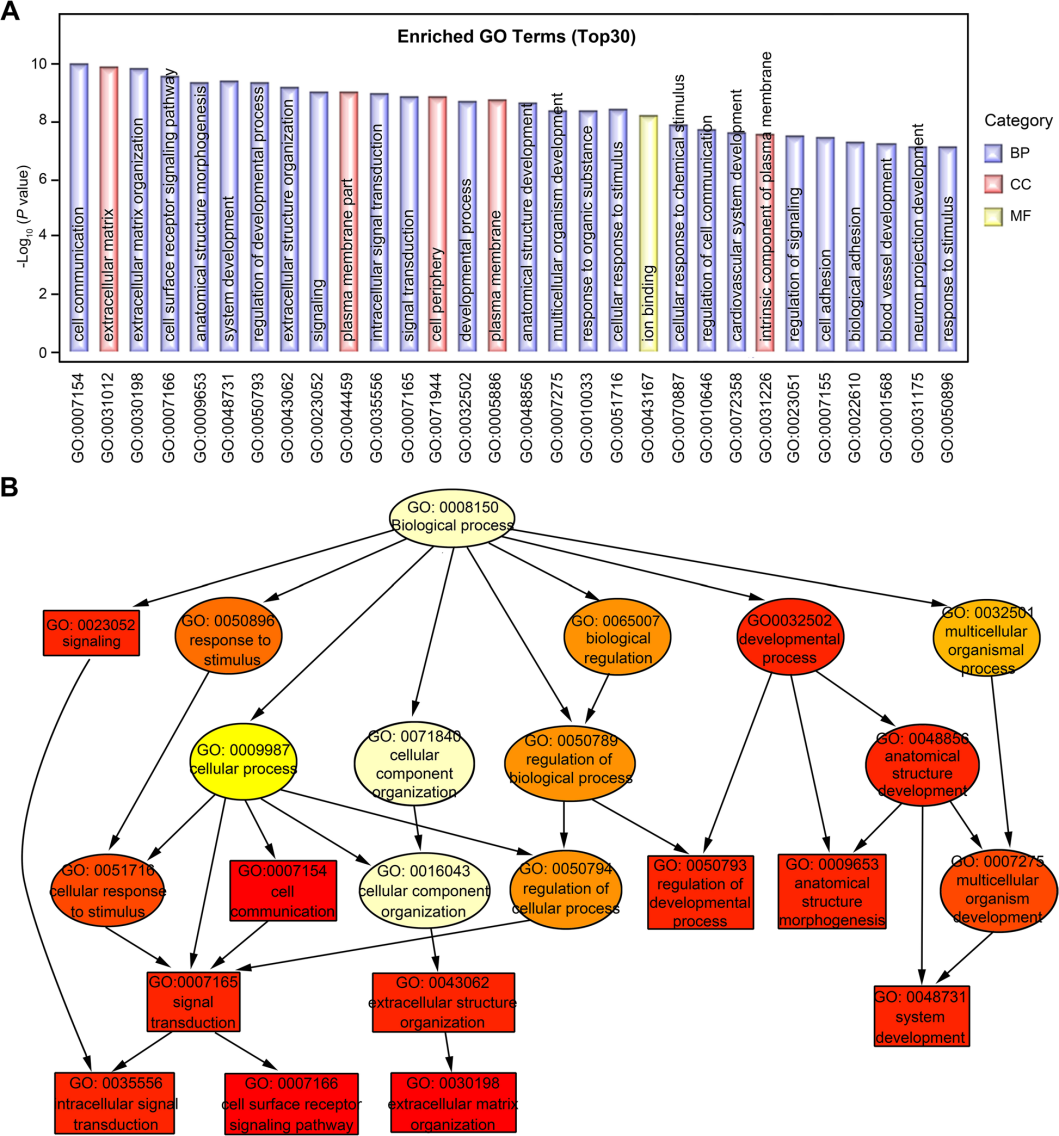
The gene ontology (GO) enrichment analysis of differential genes. **A** The top 30 enriched GO terms were shown to identify the potential function of differential genes in biological processes (BP), cellular component (CC) and molecular functions (MF). **B** Directed acyclic graph (DAG) of the enriched GO terms. The ID of GO terms and description were presented. Branch represents containment relationship. Color depth represents the degree of enrichment. The closer to red represents more significant. Rectangles represent the top 10 enriched GO terms, and others were shown as ellipse

GO analysis was performed to determine gene and gene product enrichment. Three categories were covered, including biological processes (BP), cellular components (CC) and molecular functions (MF). The top 30 enriched GO terms are shown in Fig. 6A, with 24 BP terms, 5 CC terms and 1 MF term, which indicated that downregulation of ATBF1 in MCF7 cells mainly affected biological processes. The directed acyclic graph (DAG) was used to present the enriched GO terms of the differentially expressed genes (Fig. 6B). From GO analysis of the top enriched GO terms and the DAG, we found that ATBF1-related genes mainly participated in three biological processes: signal transduction, extracellular structure organization and system development.

**Fig. 7:**
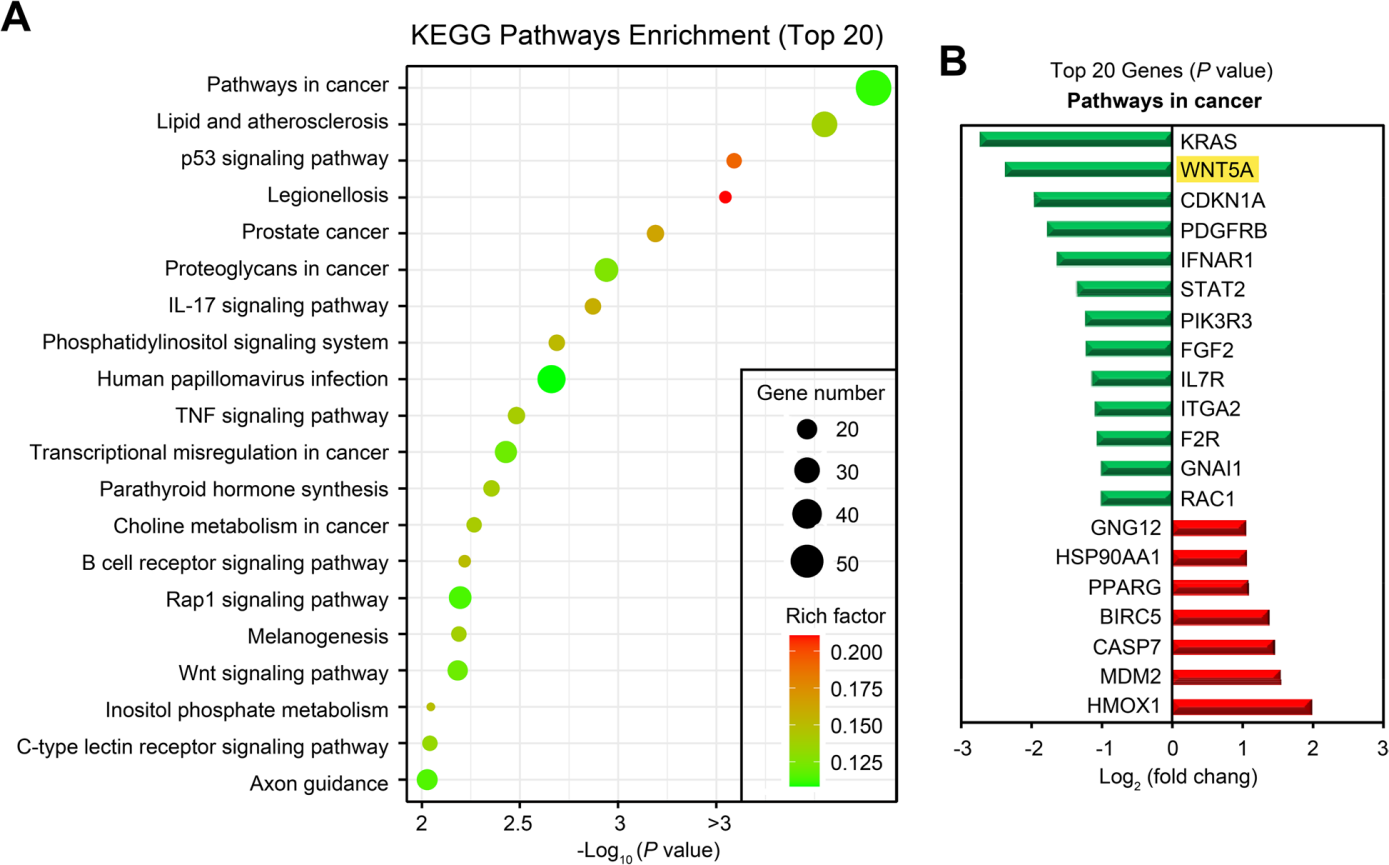
The kyoto encyclopedia of genes and genomes (KEGG) of enrichment analysis of differential genes and the essential downstream genes regulated by ATBF1. **A** The top 20 enriched KEGG pathways as shown by bubble chart. The size of bubbles represents gene number involved, and the color represents rich factor. Pathways in cancer ranked first in all enriched KEGG pathways. **B** The top 20 genes involved in pathways in cancer were ranked by *P* value

Kyoto Encyclopedia of Genes and Genomes (KEGG) analysis was performed to detect the potential pathways associated with the identified DEGs. Fisher’s exact test was used to calculate the significance of KEGG enrichment to determine ATBF1-related metabolism and signaling pathways. A bubble scatter diagram (Fig. 7A) shows the top 20 enriched KEGG pathways of differentially expressed genes, and the most important pathway influenced by ATBF1 was “Pathways in cancer”. Moreover, the top 20 genes in “Pathways in cancer” were ranked by *P* value, with 13 downregulated genes and 7 upregulated genes(Fig. 7B).

### WNT5A is an essential downstream gene of ATBF1

**Fig. 8:**
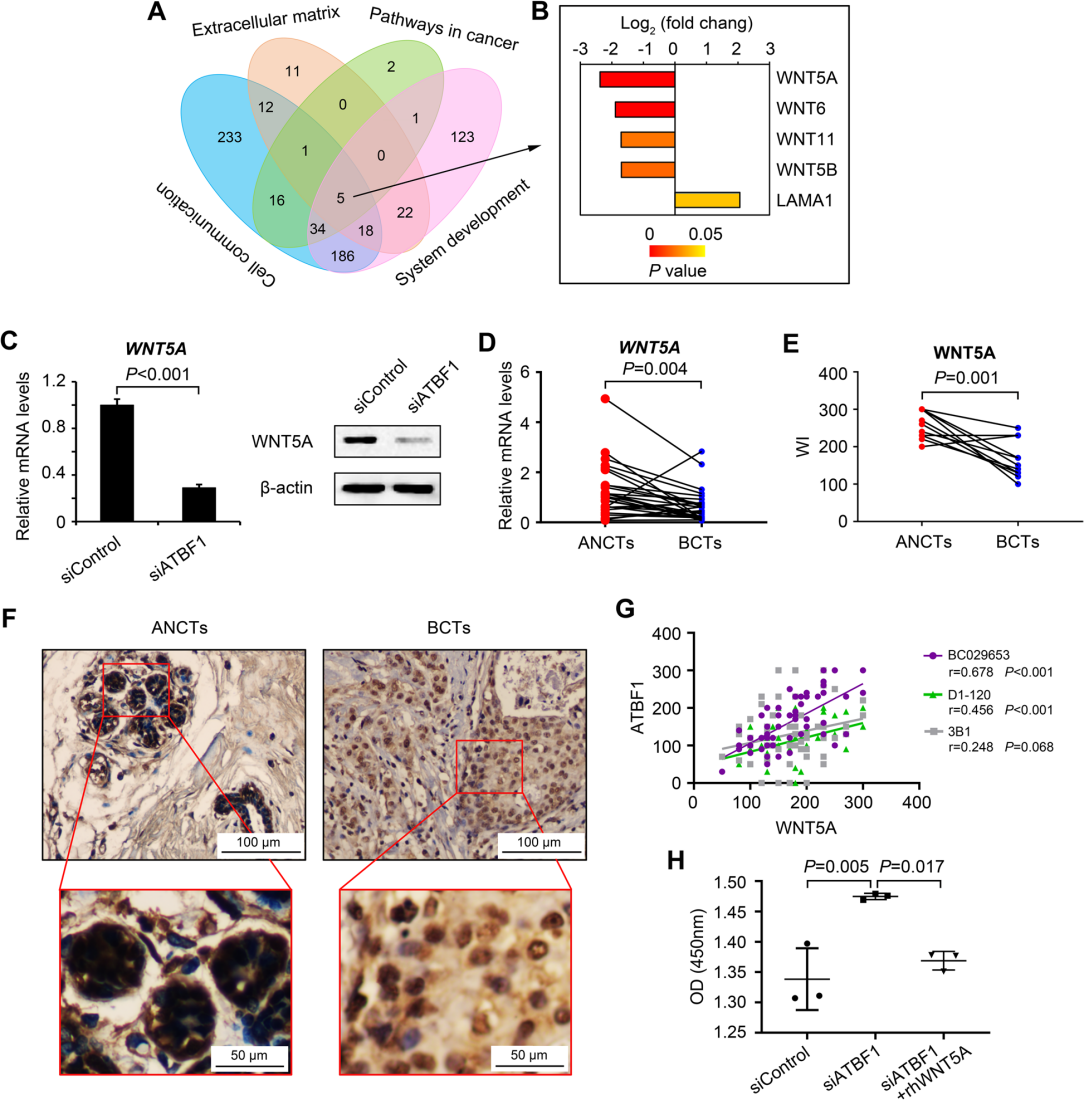
WTN5A was a crucial downstream gene of ATBF1. **A** Venn diagram of genes involved in the top 3 enriched GO (cell communication, extracellular matrix and system development) and the top 1 enriched KEGG (pathways in cancer). Five common genes (WNT5A, WNT6, WNT11, WNT5B and LAMA1) were detected. **B** The five common genes were listed by the fold change. The color represents the *P* value. **C** The expression of WNT5A in MCF7 cells transfected with siControl or siATBF1 was examined by real-time qPCR and western blot. Down-regulation of WNT5A was observed after ATBF1 silencing. Triplicate experiments were performed and *P*-value between two groups was calculated by student’s *t* test. **D** The mRNA expression of *WNT5A* in paired breast cancer cases, as determined by real-time qPCR. **E** The protein levels of WNT5A in paired breast cancer cases, quantified by WI index. Paired *t* test was used to calculate *P*-value in Fig. 8D&E. **F** Representative IHC images of WNT5A in paired breast cancer cases. **G** The correlation between the expression of WNT5A and ATBF1 at protein level, quantified by WI index. **H** Cell proliferation assay. MCF7 cells were transfected with siControl or siATBF at the concentration of 150 nM. Twenty four hours later, the cells were treated with 200ng/mL recombinant human WNT5A to rescue the down-regulation of WNT5A induced by ATBF1 knockdown. The cell number was determined as OD450 after 24 h of WNT5A treatment with a cell counting kit. One-way ANOVA with Bonferroni was used to determine the statistical differences among the three groups

VENNT2.1 (https://bioinfogp.cnb.csic.es/tools/venny/) software was used to analyze the DEGs with the most highly enriched GO terms (cell communication, extracellular matrix and system development) and KEGG pathways (pathways in cancer) (Fig. 8A). A total of 505 differentially expressed genes were associated with cell communication, 69 differentially expressed genes were associated with the extracellular matrix, 389 differentially expressed genes were associated with system development, and 59 differentially expressed genes were associated with pathways in cancer. Most importantly, 5 genes were found to be associated with all of the most highly enriched GO terms and KEGG pathways: WNT5A, WNT6, WNT11, WNT5B and LAMA1 (Fig. 8B), which suggested that WNTs may be essential targets of ATBF1 and may contribute to breast cancer tumorigenesis. Moreover, WNT5A was determined to be the crucial downstream gene with the highest expression change and the lowest *P* value (Fig. 8B).

The reduced expression of WNT5A regulated by ATBF1 was further confirmed by real-time qPCR at the mRNA level and by western blotting at the protein level in MCF7 cells (Fig. 8C). We also detected the expression of WNT5A in breast cancer patients. As shown in Fig. 1, ATBF1 was downregulated in BCTs compared with ANCTs. Consistently, the expression of WNT5A was also significantly lower in BCTs than in ANCTs in paired cases at both the mRNA (Fig. 8D) and protein levels (Fig. 8E). The protein expression of WNT5A was assessed by IHC staining, as shown in Fig. 8F. A significant correlation was found between the protein expression of ATBF1 and WNT5A in breast cancer cases when ATBF1 was recognized with BC029653 (Fig. 8G, *r* = 0.678, *P* < 0.001) and D1-120 (Fig. 8G, *r* = 0.456, *P* < 0.001). However, no significant correlation was detected between the C-terminus of ATBF1 (3B1) and WNT5A in breast cancer cases (Fig. 8G, *r* = 0.248, *P* = 0.068).

In addition to the expression correlation between ATBF1 and WNT5A, we further investigated whether ATBF1 regulated the proliferation of breast cancer cells via WNT5A. After transfection of siATBF1 in MCF7 cells, WNT5A expression was reduced (Fig. 8C), and the cell number was increased with higher OD absorbance than that in the control group (Fig. 8H). Then, recombinant hWNT5A was used to rescue the expression of WNT5A, and the promotion of cell proliferation induced by siATBF1 was inhibited (Fig. 8H).

## Discussion

ATBF1 is the largest transcription factor known to date and plays essential functions in both physiological and pathological processes. Mutations, deletions and single nucleotide polymorphisms (SNPs) of ATBF1 were correlated with multiple diseases, including cancers [5, 9, 10, 22, 29] and cardiovascular diseases [30, 31]. ATBF1 has been demonstrated to be a potential diagnostic marker for skin cancer, prostate cancer, gastric cancer, bladder cancer and non-small cell lung cancer (NSCLC) [9, 12, 13, 32]. Kawaguchi et al. [13] reported that ATBF1 staining was an independent prognostic factor for intravesical recurrence-free survival after adjusting for cellular grading and pathological staging, which contributed to the prediction of superficial urothelial bladder carcinoma at initial transurethral resection. Zhang et al. [9] reported that ATBF1 mutations were a novel predictive biomarker for NSCLC patients receiving immune checkpoint inhibitor treatment; they were positively correlated with known immunotherapy response biomarkers, including immune-related gene expression and T-cell infiltration biomarkers.

In the present study, we collected 44 fresh-frozen tissues (31 ANCTs and 44 BCTs) for mRNA expression analysis via real-time qPCR and 80 formalin-fixed and paraffin-embedded tissues (40 ANCTs & 80 BCTs) for protein analysis via IHC staining with 3 independent anti-ATBF1 antibodies. We found that the expression of ATBF1 at both the mRNA and protein levels in BCTs was significantly lower than that in ANCTs, and this difference was more apparent and significant in paired cases. The reduced expression of ATBF1 was consistent with that in other cancers, such as prostate cancer and gastric cancer, which suggested ATBF1 as a tumor suppression gene.

Tumor size, lymph node metastasis, histological grade, and ER, PR and HER2 status are the main diagnostic determinates used in routine clinical practice. To further confirm the potential diagnostic role of ATBF1 in the clinic, the correlations of ATBF1 expression with these parameters were analyzed. Consistent with previous studies [24], ATBF1 protein expression was not correlated with tumor size, lymph node metastasis, ER status or PR status. However, a significant correlation was found between ATBF1 protein levels and histological grade. Histological grade has been demonstrated to be an important factor of breast cancer outcome that can accurately predict tumor behavior, particularly in earlier small tumors [33]. However, the current method for the determination of histological grade requires a trained pathologist to analyze hematoxylin-eosin (HE)-stained tumor tissue sections. The stages are subjective to a certain extent, and sometimes the histological grades of HE sections are difficult to distinguish. By comparison, specific protein staining visualized with brown coloration should be more precise.

Moreover, histological grade is based on the degree of differentiation of the tumor tissue [34]. Higher histological grade represents lower differentiated status with poor prognosis. The expression of ATBF1 in stage I/II tumors was higher than that in stage III tumors, which indicated that ATBF1 levels were positively correlated with cell differentiation. Consistently, many studies have demonstrated that ATBF1 participates in cell differentiation and tissue development in various tissues. Ido et al. [35] first reported the function of ATBF1 in neuronal cells and brain development. The expression of *ATBF1* mRNA was induced at an early stage during neuronal differentiation with retinoic acid in P19 mouse embryonal carcinoma cells and declined at a later stage. However, when P19 cells underwent muscle differentiation with dimethyl sulfoxide treatment, no comparable level of *ATBF1* mRNA was detected. Furthermore, ATBF1 was demonstrated to promote the survival of neurons by inducing the expression of platelet-derived growth factor receptor β (PDGFRB) [36]. Recently, ATBF1 was reported to play essential roles in mammary development via hormone-hormone receptor pathways. In ATBF1 knockout mice, ductal elongation and bifurcation were enhanced during puberty [14], while lactogenesis was interrupted during lactation along with great reduction in the size and number of alveoli [16]. In breast cancer patients, the degree of cell differentiation seriously affects the treatment plan and tumor prognosis. The significant correlation between ATBF1 protein level and histological grade (cell differentiation) suggested that ATBF1 might be a useful biomolecular marker for breast cancer.

Interestingly, as a transcription factor, ATBF1 was localized in nuclei in normal cells but was transferred to the cytoplasm in head and neck, gastric, skin and bladder cancer cells. Here, we also found that ATBF1 was mainly localized in nuclei in ANCTs, whereas it was localized in the cytoplasm in BCTs. Kawaguchi et al. [13] identified three new NLSs (1387–1400, 2947–2959 and 2987–3005) in addition to the NLS (2615–2617) reported by Sun et al. [37]. They found that the mislocalization of ATBF1 in bladder carcinomas was caused by the cleavage of the ATBF1 protein into fragments. The fragments in the middle region containing nuclear localization signals (NLSs) were localized in nuclei, and the fragments in the N-/C-terminal ends lacking NLSs were localized in the cytoplasm [13]. However, in this study, this phenomenon was not detected in breast cancer tissue samples. All three antibodies recognized the N-terminal end, middle region and C-terminal end, indicating that the ATBF1 protein was mainly localized in the cytoplasm in breast cancer tissues. Unfortunately, the in-depth mechanism of the role ofATBF1 in breast cancer was not clarified in the present study. However, a few signaling pathways have been reported to lead to the translocation of ATBF1. In ER-positive breast cancer cells, estrogen is a possible molecule that induces the translocation of ATBF1 from the nucleus to the cytoplasm in an ER-dependent manner, as we reported previously [38], and Mabuchi et al. [6] reported TGF-β signal transduction as another possible mechanism. ATBF1 nuclear localization was significantly correlated with runt domain transcription factor 3 (RUNX3) in gastric cancer tissue samples, and ATBF1 was transferred from the cytoplasm to the nucleus under TGF-β treatment in SNU16 gastric cancer cells, which was also detected in HaCaT cells [25].

In addition, the expression of ATBF1 detected by distinct antibodies was variable. While D1-120 seemed to have the strongest reduction in weight index in BCTs, the other antibodies from the N- and C-termini of ATBF1 seemed to have subtle changes. A few possible explanations for this finding were considered. First, there are two isoforms of ATBF1, ATBF1-A and ATBF1-B [39]. ATBF1-A is a large transcription factor with a molecular weight of 404kD, while ATBF1-B lacks 914 amino acids in the N-terminus with a molecular weight of 306kD. The functions and expression levels of ATBF1-A and ATBF1-B vary in different cells [8, 39]. ATBF1-A has been shown to be the main form expressed in most tissues [40], and recent studies have mainly focused on ATBF1-A [19, 37, 41]. Whether the two isoforms exist in cancer tissues is currently unexplored. Moreover, ATBF1 might be cleaved into several fragments in carcinomas. In urothelial bladder carcinoma, Kawaguchi et al. [13] found that ATBF1 was cleaved into small fragments with or without nuclear localization signals (NLSs), which led to abnormal subcellular localization of ATBF1. Kataoka et al. [8] reported a similar phenomenon of ATBF1 expression and subcellular localization in colon tumors with 4 distinct antibodies. In the present study, although the subcellular localization of ATBF1 was not observed by detection with distinct antibodies, the expression index (WI) of ATBF1 was different, which may have been caused by cleavage or degradation. Dong et al. [20] found that estrogen-responsive finger protein (EFP) mediated estrogen-induced ATBF1 protein degradation. They designed several fragments of ATBF1 to clarify the exact regions of the interactions between EFP and ATBF1. The region 2107–2147 recognized by D1-120 was demonstrated to interact with EFP and to mediate degradation by EFP [20] but the regions recognized by the other two antibodies did not exhibit this phenomenon.

Although ATBF1 was initially considered a candidate tumor suppressor gene in prostate cancer [10] and was further identified in breast cancer [18, 24], a recent study demonstrated that ATBF1 acts as a protumorigenic gene in ER-positive breast cancer cells by enhancing stem-like features [42]. The reasons for such inconsistencies are likely very complicated [42]. There are a few possible explanations for this phenomenon. First, the two isoforms of ATBF1, ATBF1-A and ATBF1-B, might function conversely in different cells [43]. For example, in HuH-1 and HuH-7 hepatoma cells, both ATBF1-A and ATBF1-B strongly inhibited the enhancer activity of *AFP*. In HepG2 hepatoma cells, ATBF1-A inhibited the enhancer activity of *AFP*, while ATBF1-B promoted its activity [43]. Then, the culture medium of ER-positive cells might be essential for the transformation of ATBF1 function [42]. ATBF1 inhibited estrogen-mediated cell proliferation in a hormone-free medium when only estrogen signaling was activated [18]. In contrast, ATBF1’s pro-proliferation function was dominant when cells were cultured in complete medium with activation of both estrogen and progesterone signaling [42]. Here, we noticed that Dong et al. [42] cultured MCF7 cells in DMEM, while MCF7 cells were cultured in MEM in the present study. Different components in the medium may transform the function of ATBF1, especially the high glucose in DMEM. Xie et al. [44] reported that high levels of glucose triggered PTEN neddylation and induced PTEN nuclear import and that nuclear neddylated PTEN stabilized fatty acid synthase to promote tumor development, which indicated that neddylation switched PTEN from a tumor suppressor to a tumor promoter. In addition, SUMOylation induced by high glucose is another possible explanation for switch in ATBF1 function. Zhou et al. [45] found that high glucose induced SUMOylation of Smad4 via SUMO2/3 and activated TGF-β/Smad signaling in mesangial cells. SUMOylation of IGF-1R in periodontal ligament stem cells cultured in high glucose medium inhibited osteogenic differentiation [46]. Lysine 2806 is an important site for ATBF1 SUMOylation, whose mutation prevents MDA-MB-231 cells from forming tumors in nude mice [21]. Whether high levels of glucose in DMEM trigger the switch in ATBF1 function is uncertain; however, it is a plausible explanation and needs further investigation.

Most importantly, we discovered a novel essential gene (WNT5A) regulated by ATBF1 via a microarray assay. Enriched GO analysis showed that system development was one of the most crucial biological processes affected by ATBF1, which was consistent with previous studies. The other two important biological processes were signal transduction and extracellular structure. After overall analysis of the most highly enriched KEGG pathways (pathways in cancer) and GO terms, WNT5A was found to be an attractive mechanism of ATBF1’s role in breast cancer, with the highest *P* value and fold change of all identified DEGs. WNT5A signaling is critical for normal developmental processes, including adhesion, proliferation, differentiation, migration and polarity. Aberrant activation or inhibition of WNT5A signaling exerts both oncogenic and tumor suppressive effects [47]. WNT5A has been demonstrated to inhibit the cell growth, migration and invasiveness of thyroid and colorectal cancer cells [48, 49], while increased WNT5A expression is involved in the aggressiveness of other types of cancers, such as melanoma [50] and gastric cancer [51]. In breast cancer, WNT5A is considered a tumor suppressor since loss of WNT5A is associated with poor prognosis [52]. In the present study, we also demonstrated that the expression of WNT5A protein was reduced in BCTs compared with that in ANCTs via IHC staining, which was significantly correlated with the expression of ATBF1 protein. However, no significant correlation between WNT5A and ATBF1 at the mRNA level was observed, which might be caused by the discrepancy between WNT5A mRNA and protein levels [53].

## Conclusion

ATBF1 acts as a tumor suppressor gene in malignant tumors via multiple aspects, including mutations, chromosome deletion, transcriptional downregulation and protein mislocalization. ATBF1 was also reported to play an essential role in multiple signaling pathways to regulate cell proliferation, differentiation and apoptosis. In the present study, we demonstrated the reduced expression of ATBF1 in BCTs and mislocalization from the nucleus in ANCTs to the cytoplasm in BCTs. The correlation between ATBF1 expression and histological grade suggests that ATBF1 is a potential diagnostic marker of breast cancer that reflects cell differentiation and can be used to help direct malignancy therapy. Microarray analysis revealed that ATBF1 mainly functions in signal transduction, extracellular structure organization, system development and pathways in cancer. Most importantly, WNT5A was detected as a crucial downstream gene of ATBF1 via a bioinformatics assay, which was further confirmed in MCF7 cells and clinical samples. This study provides another perspective to investigate the function of ATBF1 in breast epithelial cell activities and tumorigenesis.

## Supplementary Information

**Additional file 1.**

## Data Availability

The original data supporting these findings are available at any time upon request to the corresponding author. The datasets of RNA-seq generated and analyzed during the current study are available in the NCBI repository (SRA accession numbers for control siRNA: SRR21020962, SRR21020963, SRR21020964; SRA accession numbers for ATBF1 siRNA: SRR21020959, SRR21020960, SRR21020961) (Web links: https://www.ncbi.nlm.nih.gov/Traces/study/?acc=SRP391173&o=acc_s%3Aa). Other data are not publicly available due to privacy and ethical considerations.
