# Supplementary material for: ATBF1 is a potential diagnostic marker of histological grade and functions via WNT5A in breast cancer
Source: BMC Cancer. 2022 Dec 7;22:1280. doi: 10.1186/s12885-022-10380-2 (PMC9727999; doi:10.1186/s12885-022-10380-2)
Supplement: Supplementary file 1 — Additional file 1. [file 12885_2022_10380_MOESM1_ESM.docx]

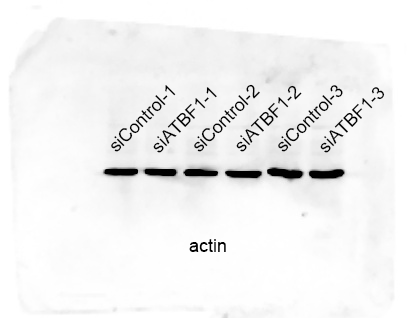

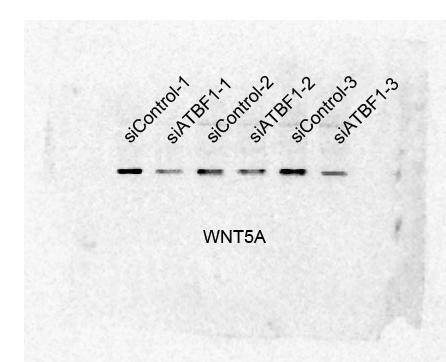


Original data of WNT5A western blot. WNT5A and β-actin protein levels in control siRNA group (siControl) or ATBF1 siRNA group (siATBF1) were assessed by western blot. β-actin were introduced as an internal control.


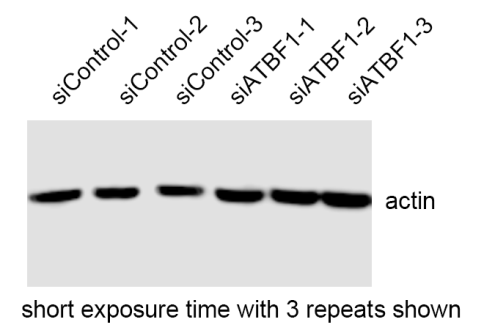

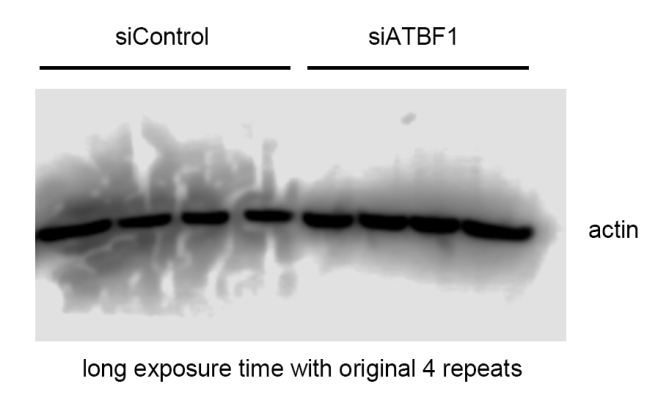

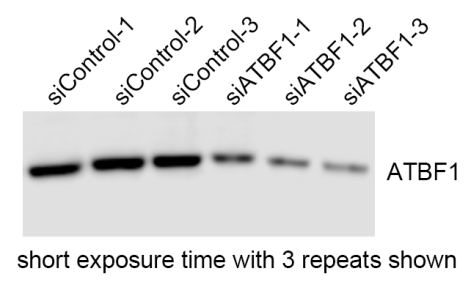

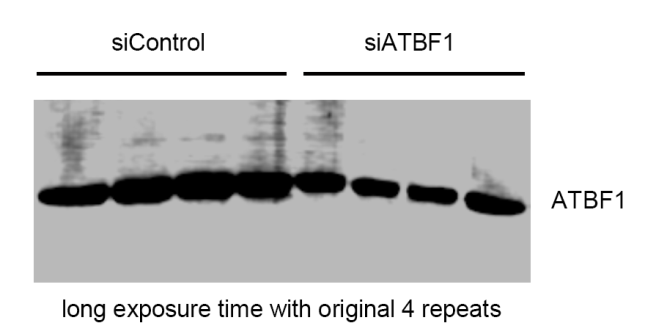


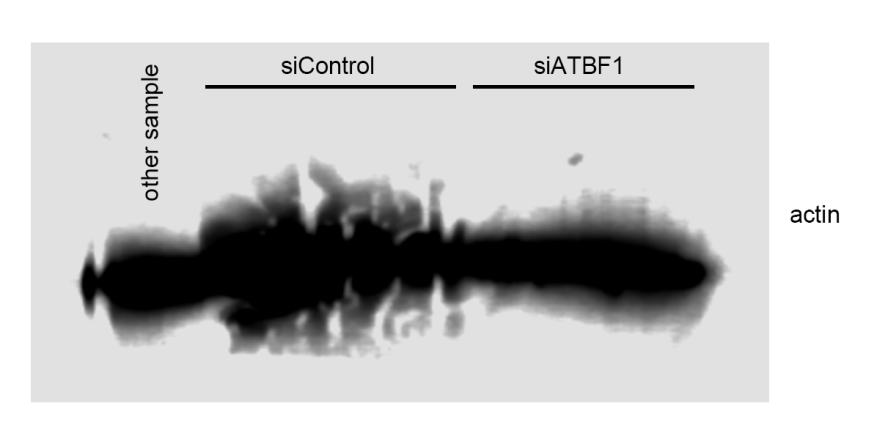


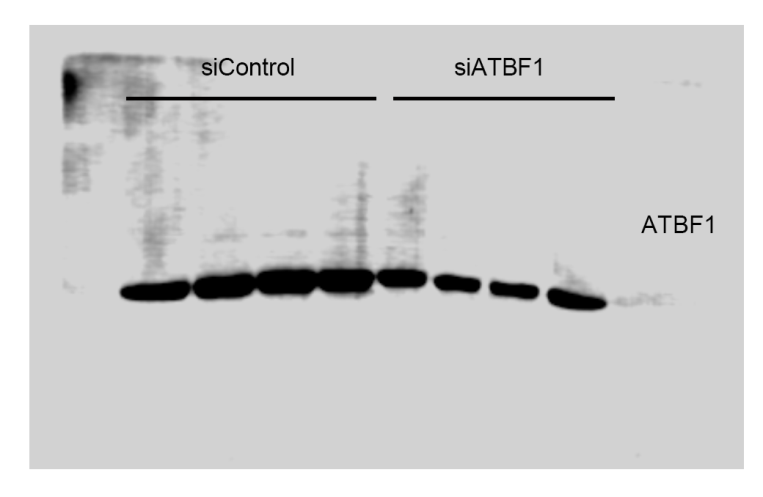


MCF7 were tranfected with ATBF1 siRNA (siATBF1) or control siRNA (siControl) at the final concentration of 150 nM. The protein levels of ATBF1 were examined by western blots. The proteins were separated by 10% SDS-PAGE for actin detection and 6% SDS-PAGE for ATBF1 detection. The blots were cut prior to hybridization with antibodies to save antibodies. Even with the longest exposure time, the membrane edges were not visible for actin detection.
